# Firearm Storage and Carrying Practices and Suicidal Behaviors in US Army Service Members

**DOI:** 10.1001/jamanetworkopen.2026.8268

**Published:** 2026-04-21

**Authors:** Catherine L. Dempsey, James C. West, Claire Houtsma, Jingning Ao, Robert Bossarte, Matthew K. Nock, Kelly L. Zuromski, Matthew W. Georg, Katy Haller, Deborah Probe, Luke L. Sumberg, David M. Benedek

**Affiliations:** 1 Center for the Study of Traumatic Stress, Department of Psychiatry, Uniformed Services University of the Health Sciences, Bethesda, Maryland; 2 Henry M. Jackson Foundation for the Advancement of Military Medicine, Inc, Bethesda, Maryland; 3 Southeast Louisiana Veterans Health Care System, South Central Mental Illness Research, Education and Clinical Center, New Orleans; 4 Department of Psychiatry and Behavioral Neuroscience, University of Southern Florida, Tampa; 5 Department of Psychology, Harvard University, Cambridge, Massachusetts

## Abstract

**Importance:**

Suicide is a critical public health crisis for military personnel; firearms are the predominant suicide method among military service members.

**Objective:**

To examine associations of firearm storage and carrying behaviors with suicidal behaviors within a large, cross-sectional sample of US Army soldiers who own personal firearms, after controlling for known suicide risk factors.

**Design, Setting, and Participants:**

This cross-sectional study used data from participants in the Army Study to Assess Risk and Resilience in Servicemembers–Longitudinal Study Wave 2 (STARRS-LSWS) survey (collected April 2018 to July 2019). Analyses were conducted from June 2022 through January 2025.

**Main Outcome and Measures:**

Self-reported suicidal behaviors were assessed with the Columbia-Suicide Severity Rating Scale. Firearm storage and carrying practice survey items were created for the STARRS-LSW2. Stressful life events items were adapted from the Life Events Questionnaire and the US Department of Defense Health-Related Behaviors Survey. Sociodemographic, Army career, and mental health disorders data were obtained from the military medical record. Multivariable logistic regression examined 30-day, 12-month, and lifetime suicidal ideation and suicide attempt. Analyses used weighted samples to account for oversampling, underrepresentation, and nonresponse.

**Results:**

The total sample of 12 022 soldiers consisted of 6561 firearm owners (54.5%) and 5461 soldiers who did not own firearms (45.4%). Participants were primarily male (10 096 [83.6%]), with a mean (SD) age of 32 (7) years. Unsecured firearm storage (loaded and unlocked) compared with secure storage (eg, unloaded) was associated with increased suicide ideation risk across all time frames: 30-day (odds ratio [OR], 1.63; 95% CI, 1.27-2.10), 12-month (OR, 1.61; 95% CI, 1.27-2.03), and lifetime (OR, 1.44; 95% CI, 1.22-1.70). Firearm owners with unsecured storage were more likely to report a suicide attempt in the past 12 months (OR, 4.93; 95% CI, 1.80-13.51). Carrying a weapon other than a firearm (eg, a knife, mace, or a club) was also associated with increased suicide attempt risk in the past 12 months (OR, 10.42; 95% CI, 2.89-37.54).

**Conclusions and Relevance:**

In this retrospective cross-sectional study, unsecured firearm storage practices were significantly associated with increased suicide risk across all time frames, after controlling for lifetime mental health disorders and history of stressful life events. Carrying a weapon other than a firearm was also associated with heightened suicide risk. The findings suggest firearm storage and carrying behavior of any weapon, not just firearms, may indicate heightened suicide risk among military service members.

## Key Points

**Question:**

Are firearm storage and carrying practices and carrying of other weapons associated with suicidal behaviors in military service members?

**Findings:**

This cross-sectional study of 6561 firearm-owning US Army soldiers found that carrying firearms, as well as other weapons generally, was associated with greater risk of suicide, as was storing a firearm unsecured (loaded and unlocked) vs unloaded.

**Meaning:**

The findings of this cross-sectional study suggest that firearm storage behavior and carrying behavior of weapons in general, not just firearms, may serve as indicators of heightened suicide risk among military service members, indicating interventions should target all weapons.

## Teaser

This cross-sectional study assesses associations of firearm storage and carrying behaviors with self-reported suicidal ideation and attempt among US Army soldiers.

## Introduction

Suicide deaths represent a serious public health problem, particularly among military service members. In 2023, the suicide rates among US Army active-duty soldiers^1^ rose to 34.8 per 100 000 and remained high compared with age- and sex-adjusted civilian rates.^2^ Firearms are the most common method, used in more than 65% of US Army active-duty service members’ suicide deaths,^1^ compared with 50% in the general population.^3^ Extant research has found that the presence of a firearm in the home is associated with greatly increased risk of death by suicide,^4^ suggesting that firearm access plays a crucial role. This elevated risk may be compounded by higher firearm ownership rates among military service members and veterans (approximately 50%),^5,6^ compared with about 38% among general-population adults.^7^

Although firearm ownership is not believed to prompt suicidal ideation,^8^ ready access to a firearm may heighten risk when suicidal ideation develops. To that end, there is some evidence to suggest that risk of suicide-related outcomes is further increased when firearms are easily accessible within the home, such as when stored in an unlocked location and/or loaded. For example, within a nonclinical sample of military service members who were firearm owners, lifetime suicidal ideation was associated with storing firearms in an unlocked location and loaded.^5^ Similarly, service-member firearm owners with a lifetime history of suicidal ideation or recent thoughts of death or self-harm had increased odds of storing firearms either unlocked, loaded, or both unlocked and loaded.^9,10^ Furthermore, informant reports of US Army suicide decedents showed 4 times greater likelihood of storing a personal firearm loaded and 3 times greater likelihood of carrying a personal firearm compared with living propensity-matched controls.^11^

A broad range of factors contribute to suicidal thoughts and behaviors among soldiers, and our understanding of the transition from suicidal ideation to attempt is improving. For example, medically documented suicidal ideation is most common among enlisted soldiers early in their military service, during their first tour of duty^12^; firearm suicide attempts are more likely to occur among US Army soldiers who previously deployed compared with those who have never deployed^13^; suicide attempt is more prevalent among combat engineers and combat medic occupational specialties^14^; recent onset of suicidal ideation, presence of a suicide plan, low controllability of suicidal thoughts, and extreme risk taking are all associated with increased likelihood of a suicide attempt^15^; and soldiers who experience relationship problems, military punishment, and perceived failure are more likely to die by suicide than soldiers with suicidal ideation.^16^ Despite these known risk factors, the relationship of firearm storage and carrying practices, as well as carrying behaviors for other weapons (eg, a knife, mace, or a club), with suicide risk remains understudied.

The current study sought to extend previous research by examining associations between firearm storage and carrying behaviors and suicidal ideation and attempts within a large longitudinal sample of US Army soldiers who own personal firearms, while accounting for established risk factors. We hypothesized that unsecured firearm storage (ie, loaded and unlocked) and firearm carrying would be associated with significantly higher odds of suicidal ideation and suicide attempts, even after controlling for established risk factors for suicide. Results consistent with these hypotheses would strengthen our understanding of the risks associated with certain firearm practices and may help identify individuals for targeted suicide prevention interventions.

## Methods

### Sample

The sample for this cross-sectional study consisted of data from the Army Study to Assess Risk and Resilience in Servicemembers–Longitudinal Study Wave 2 (STARRS-LSW2),^17^ which is a multiwave survey of eligible active-duty soldiers who had previously taken a baseline survey as part of the Army STARRS New Soldier Study or All Army Study or Pre-Post Deployment surveys^18,19^ and consented to linkage to US Army–Department of Defense (DoD) administrative records. In STARRS-LSW2 (April 2018 to July 2019), the survey consisted of soldiers and veterans who completed the interviews and consented to administrative linkage. The Army STARRS-LSW2 study procedures were approved by the institutional review board of the University of Michigan Institute for Social Research, with secondary review and approval by the Uniformed Services University, University of California–San Diego, and Harvard Medical School. This study followed the Strengthening the Reporting of Observational Studies in Epidemiology (STROBE) reporting guideline. For this study, the Uniformed Service University institutional review board determined that approval and consent were not required because secondary analysis of the deidentified data in STARRS-LSW2 did not constitute human participant research.

The final analytic sample for this study consisted of respondents who consented to administrative linkage and who provided information for all measures used in the current study. STARRS-LSW2 oversampled Army STARRS baseline participants with a history of mental disorders or suicidality, women, National Guard and Reserve members, and Special Operations soldiers. Earlier reports provide more details regarding sampling, weighting, and other STARRS-LSW2 procedures.^20,21^ The weights incorporate nonresponse and poststratification adjustments for Army STARRS baseline and STARRS-LSW2 follow-up survey data as well as adjustments to account for oversampling of baseline respondents with certain characteristics and underrepresentation of difficult-to-recruit participants in STARRS-LSW2.^18,19,20^

### Measures

The STARRS-LSW2 survey assessed a wide range of risk and protective factors for suicide; those relevant to this study are described in the following sections. Development of the measures and details of the scales are described elsewhere.^21^

#### Demographics

Sociodemographic information (sex, age, marital status, race and ethnicity, and Army career characteristics) came from a combination of administrative records supplemented by STARRS-LSW2 interview responses. Administrative records were pulled from the month-year record when the survey was taken or the last administrative record for participants who had left service. Race and ethnicity, ascertained from the administrative record and included in the analysis as covariates, were coded as Asian or other (American Indian or Alaska Native, Native Hawaiian, or Other Pacific Islander), Hispanic, non-Hispanic Black (hereafter, Black), or non-Hispanic White (hereafter, White).

#### Psychiatric Disorders

Military medical records provided a history of 22 psychiatric diagnoses by *International Classification of Diseases, Ninth Revision (ICD-9)* codes and counts of inpatient and outpatient encounters. The mental health variable included attention-deficit/hyperactivity disorder, adjustment disorder, alcohol abuse disorder, anxiety, bipolar disorder, conduct or oppositional defiant disorder, minor depression, major depressive disorder, eating disorder, nonaffective psychosis, organic mental disorders, other mental disorders or mental illnesses, other impulse-control disorders, personality disorders, sex disorders, sleep disorders, somatoform or dissociative disorders, traumatic stress, posttraumatic stress disorder (PTSD), drug-induced mental illness, drug abuse without dependence, and drug dependence.

#### Firearms Storage and Carrying Behaviors

Respondents who reported owning 1 firearm were asked whether that firearm was unlocked, loaded, both, or neither. Respondents owning more than 1 firearm were asked how many of those were loaded and whether they were loaded and unlocked. If individuals owned multiple firearms and more than 1 of those firearms were loaded, they were asked how many were unlocked. Responses for these questions were combined into a single firearm storage variable with responses grouped into whether any of the firearms were stored loaded and locked, loaded and unlocked, or unloaded. Firearm owners were also asked, “Not counting times you are on duty, how often do you carry a firearm with you (or in your vehicle) when going out in the neighborhood (eg, going for a walk or to the grocery store)?” Response options were dichotomized into “none of the time or a little or some of the time” and “most or all or almost all of the time.”

#### Other Weapon Carrying Behaviors

To assess weapon carrying behaviors, participants were asked, “Not counting times you are on duty, how often do you carry some other weapon such as a knife, club, or mace with you (or in your vehicle) when you’re out in your neighborhood?” The same response options for firearm carrying behaviors were dichotomized for other weapon carrying.

#### Stressful Life Events

The stressful life events (SLE) items were adapted from the Life Events Questionnaire^22^ and the DoD Health-Related Behaviors Survey.^23^ The scale consists of 14 lifetime SLE and 15 deployment-related SLE. Additional details are provided in eTable 1 in Supplement 1, which provides a list of SLE codes collapsed thematically into lifetime interpersonal violence, deployment interpersonal violence, natural disaster, accident, and other (something else).

#### Suicidal Behaviors

Self-reported 30-day, 12-month, and lifetime suicide ideation and suicide attempt were assessed with the Columbia-Suicide Severity Rating Scale (C-SSRS) modified for STARRS.^24^ Dichotomous variables (yes, no) were created for past-month suicide ideation, past-year suicide ideation, lifetime suicide ideation, past-year suicide attempt, and lifetime suicide attempt. Additional details can be found in the eMethods in Supplement 1.

### Missing Values

Item-level administrative data were used to update missing demographic survey data. If there was no information, we looked at other data sources for information. Any data still missing were assigned to the reference category.

### Statistical Analysis

Statistical analysis was performed from June 2022 through January 2025 with R, version 4.4.1 (R Project for Statistical Computing), using the glmnet package for least absolute shrinkage and operator (LASSO) regression, and forest plots were generated in R, version 4.4.1, using the forestploter package.^25^ The SURVEYLOGISTIC procedure in SAS, version 9.4 (SAS Institute Inc), incorporated survey design variables.^26^ The weighted samples were used in all analyses.

Univariable logistic regression models tested the significance of each examined variable among participants with suicide ideation and suicide attempt in the firearm-owning subsample. Items tested included age; sex; rank; deployment status; race and ethnicity; marital status; age at Army entry; educational level; current military status; firearm ownership; firearm storage; firearm carrying; other weapon carrying; lifetime exposure to interpersonal violence; exposure to interpersonal violence during deployment; and lifetime mental health disorders from the military medical record. Coefficients were exponentiated in logistic models to calculate odds ratios (ORs) with 95% CIs. Wald χ^2^ tests were performed when fitting each of the logistic regression models estimating suicide ideation (30-day, 12-month, and lifetime) and suicide attempt (12-month and lifetime). *P* values were adjusted for multiple comparisons using false discovery rate (FDR) procedures.^27^ Two-sided *P* < .05 was considered significant.

Among firearm owners, multivariable logistic regression models explored associations between suicide ideation (30-day, 12-month, and lifetime), suicide attempt (12-month and lifetime), and each of the firearm behavioral items (eg, firearm storage, firearm carrying behavior, and other weapon carrying behavior), controlling for covariates. We used LASSO regression as a variable selection mechanism with 10-fold cross-validation. Variables identified included sex, military status, educational level, marital status, rank, history of mental health disorder from the military medical record, lifetime exposure to interpersonal violence, and exposure to interpersonal violence during deployment. Multicollinearity was assessed with tetrachoric and polychoric correlations of the variables and included firearm storage, firearm carrying, and other weapon carrying separately in each model while controlling for covariates.

## Results

### Demographics

The total STARRS-LSW2 sample consisted of 14 508 eligible active-duty soldiers and veterans; of those, 12 156 completed interviews and 12 022 consented to administrative linkage. The final sample consisted of 6561 firearm owners (54.5%) and 5461 participants who did not own firearms (45.4%). The sample included 10 096 males (83.6%) and 1926 females (16.4%), with mean (SD) age of 32 (7) years; 785 participants (6.8%) were Asian or other race and ethnicity, 1516 (16.1%) were Black, 1405 (11.9%) were Hispanic, and 8316 (65.2%) were White. A total of 4621 participants (42.7%) had a rank of E1 to E4 (junior enlisted), and age of entry in the Army was between 17 and 20 years for 6444 participants (55.6%); 4459 participants (37.4%) had 1 to 4 years of active service and 9424 (77.1%) had a history of prior deployment. The frequencies and weighted percentages of the whole sample are provided in eTable 2 in Supplement 1. Most gun owners (3793 [58.1%]) and participants who did not own guns (2932 [53.0%]) did not have a history of mental health disorders from the military medical record (Table 1).

**Table 1.** Demographic and Military Characteristics of STARRS-LSW2 Respondents

Characteristic	Participants, No. (weighted %) (N = 12 022)		χ^2^^a^	*P* value, FDR
	Firearm owners (n = 6561)	Not firearm owners (n = 5461)		
Sex				
Female	842 (13.1)	1084 (19.9)	122.19	<.001
Male	5719 (86.9)	4377 (80.1)		
Age of entry into Army, y				
17-20	3587 (58.1)	2857 (52.9)	45.52	<.001
21-24	1866 (26.4)	1539 (27.6)		
≥25	1108 (15.5)	1065 (19.5)		
Deployment status				
Never	1291 (21.8)	1307 (24.2)	10.31	.001
Current or previous	5270 (78.2)	4154 (75.8)		
Military status				
Active duty	1897 (27.0)	1559 (28.0)	45.24	<.001
Activated G or R or other	308 (3.8)	227 (3.0)		
G or R not currently on active duty	1381 (18.9)	967 (14.6)		
Retired, separated Army, or G or R	2975 (50.3)	2708 (54.4)		
Marriage status				
Never	4245 (63.7)	3011 (54.4)	113.70	<.001
Previous or current	2316 (36.3)	2450 (45.6)		
Race and ethnicity				
Asian or other^b^	365 (5.4)	420 (8.4)	355.50	<.001
Black	610 (12.5)	906 (19.9)		
Hispanic	590 (9.5)	815 (14.5)		
White	4996 (72.6)	3320 (57.2)		
Rank^c^				
E1-E4	2324 (39.9)	2297 (44.4)	48.61	<.001
E5-E6	2459 (34.4)	1955 (34.1)		
E7-E9	700 (9.7)	520 (9.7)		
WO or CO	1078 (16.0)	689 (11.7)		
Years of active service				
1-4	2348 (36.9)	2111 (38.0)	4.16	.13
5-10	2370 (34.7)	1853 (32.8)		
>10	1835 (28.4)	1487 (29.2)		
Mental health disorder diagnosis^d^				
No	3793 (58.1)	2932 (53.0)	27.67	<.001
Yes	2768 (41.9)	2529 (47.0)		

Abbreviations: CO, commissioned officer; FDR, false discovery rate; G, Guard; R, Reserve; STARRS-LSW2, Army Study to Assess Risk and Resilience in Servicemembers–Longitudinal Study Wave 2; WO, warrant officer.

^a^ χ^2^ Test assessed whether there was an association between ownership and each demographic variable.

^b^ Other includes American Indian or Alaska Native, Native Hawaiian, and Other Pacific Islander.

^c^ E1 to E4 indicates junior enlisted and E5 to E9, senior enlisted or noncommissioned officer.

^d^ Mental health disorder diagnoses were obtained from the military medical record.

### Univariable Analyses

Among firearm owners, storing a firearm loaded and unlocked (unsecured storage) was associated with increased 30-day (OR, 1.63; 95% CI, 1.27-2.10), 12-month (OR, 1.61; 95% CI, 1.27-2.03), and lifetime (OR, 1.44; 95% CI, 1.22-1.70) suicide ideation compared with participants who stored their firearm unloaded. Carrying a weapon other than a firearm (eg, a knife, mace, or a club) when out in the neighborhood was associated with increased risk of 30-day (OR, 1.35; 95% CI, 1.10-1.67), 12-month (OR, 1.41; 95% CI, 1.16-1.72) and lifetime (OR, 1.52; 95% CI, 1.32-1.74) suicide ideation compared with participants who never carried other weapons (eTable 3 in Supplement 1).

Firearm owners who stored their firearm unsecured were more likely to report a history of 12-month (OR, 4.93; 95% CI, 1.80-13.51) and lifetime (OR, 1.55; 95% CI, 1.04-2.32) suicide attempt compared with those who stored their firearm unloaded. Carrying a weapon other than a firearm around in the neighborhood was associated with increased risk of 12-month suicide attempt (OR, 12.04; 95% CI, 3.61-40.09) (eTable 4 in Supplement 1).

### Multivariable Analyses

**Figure 1.**
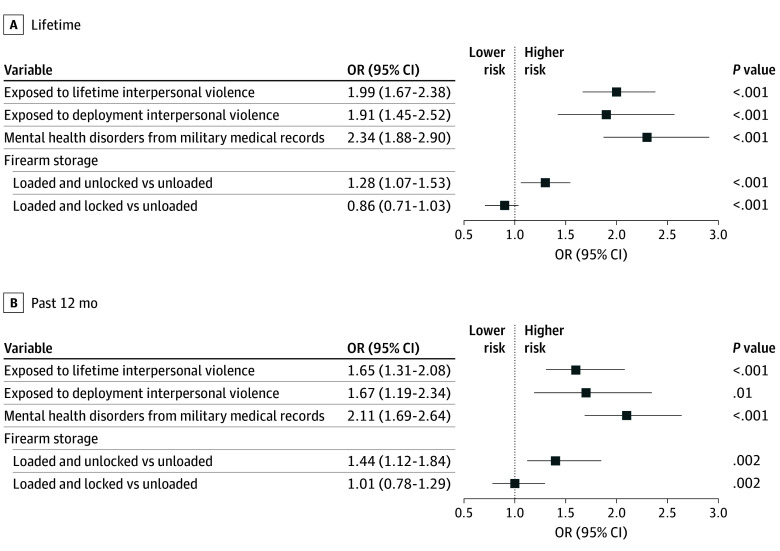
Forest Plots of Multivariable Logistic Model Results for Lifetime and 12-Month Suicide Ideation Among Firearm Owners (n = 6561) Multivariable models controlled for Army and demographic variables, including current military status, sex, marriage status, educational level, and military rank.

In multivariable models for each outcome of interest (ie, 30-day, 12-month, and lifetime suicide ideation and 12-month and lifetime suicide attempt), controlling for military status, sex, educational level, marital status, rank, lifetime exposure to interpersonal violence, exposure to interpersonal violence during deployment, history of mental health from the military medical record, and each of the firearm variables (ie, firearm storage, firearm carrying in the neighborhood, and carrying other weapons), participants who reported storing a firearm loaded and unlocked vs unloaded were more likely to report 30-day (OR, 1.48; 95% CI, 1.15-1.91), 12-month (OR, 1.40; 95% CI, 1.12-1.85), and lifetime (OR, 1.30; 95% CI, 1.06-1.54) suicide ideation (Table 2 and Figure 1). Additional details are provided in eTables 5 to 7 in Supplement 1.

**Table 2.** Multivariable Associations of Sociodemographic, Army Career, and Mental Health Characteristics With 30-Day Suicide Ideation

Characteristic	30-d Suicide ideation (N = 6561)^a^								
	Unsecured firearm storage			Firearm carrying			Carrying other weapons		
	OR (95% CI)	χ^2^	*P* value	OR (95% CI)	χ^2^	*P* value	OR (95% CI)	χ^2^	*P* value
Female sex	1.50 (1.08-2.08)	6.04	.01	1.39 (1.01-1.91)	4.12	.04	1.47 (1.06-2.04)	5.37	.02
Educational level (vs high school graduate)									
<High school or GED	1.39 (0.97-1.99)	7.47	.053	1.41 (0.98-2.04)	8.76	.03	1.37 (0.95-1.98)	7.77	.05
Some college	1.28 (0.86-1.91)			1.32 (0.89-1.96)			1.32 (0.89-1.97)		
≥College degree	0.74 (0.50-1.08)			0.72 (0.49-1.06)			0.75 (0.51-1.09)		
Current military status (vs active duty)									
Activated G or R or other	1.12 (0.57-2.18)	29.64	<.001	1.17 (0.60-2.28)	29.79	<.001	1.13 (0.58-2.20)	30.18	<.001
G or R not currently on active duty	1.43 (0.93-2.21)			1.49 (0.97-2.29)			1.48 (0.96-2.28)		
Retired, separated Army, or G or R	2.46 (1.67-3.61)			2.52 (1.72-3.69)			2.50 (1.70-3.67)		
Marriage status (vs never married)									
Currently married	0.82 (0.59-1.14)	6.16	.044	0.82 (0.59-1.14)	6.57	.04	0.81 (0.59-1.13)	6.55	.04
Previously married	1.14 (0.74-1.75)			1.17 (0.77-1.78)			1.15 (0.75-1.76)		
Rank (vs E1-E4)^b^									
E5-E6	0.90 (0.70-1.16)	4.36	.17	0.89 (0.70-1.15)	4.98	.17	0.91 (0.71-1.17)	4.41	.22
E7-E9	0.65 (0.44-0.96)			0.65 (0.44-0.96)			0.66 (0.44-0.99)		
WO or CO	0.99 (0.59-1.68)			0.96 (0.56-1.62)			0.99 (0.58-1.69)		
Exposed to interpersonal violence									
Lifetime	1.49 (1.14-1.95)	8.61	.003	1.55 (1.18-2.02)	10.03	.002	1.49 (1.14-1.95)	8.74	.003
Deployment	1.31 (0.90-1.90)	1.98	.16	1.33 (0.92-1.92)	2.33	.13	1.32 (0.91-1.90)	2.21	.14
Mental health disorder diagnosis^c^	2.29 (1.82-2.87)	50.93	<.001	2.24 (1.79-2.81)	49.66	<.001	2.22 (1.77-2.78)	48.89	<.001
Firearm storage (vs unloaded)									
Loaded and unlocked	1.48 (1.15-1.91)	8.90	.009	NA	NA	NA	NA	NA	NA
Loaded and locked	1.16 (0.87-1.55)			NA			NA		
Carrying weapons around the neighborhood all, most, or some vs none of the time									
Firearms	NA	NA	NA	0.84 (0.68-1.04)	2.58	.11	NA	NA	NA
Other (knife, mace, or club)	NA			NA	NA	NA	1.18 (0.95-1.48)	2.20	.14

Abbreviations: CO, commissioned officer; G, Guard; GED, General Educational Development; NA, not applicable; OR, odds ratio; R, Reserve; WO, warrant officer.

^a^ Firearm storage, firearm carrying, and other weapon carrying were entered into each multivariable association model separately. Analyses were weighted.

^b^ E1 to E4 indicates junior enlisted and E5 to E9, senior enlisted or noncommissioned officer.

^c^ Mental health disorder diagnoses were obtained from the military medical record.

**Figure 2.**
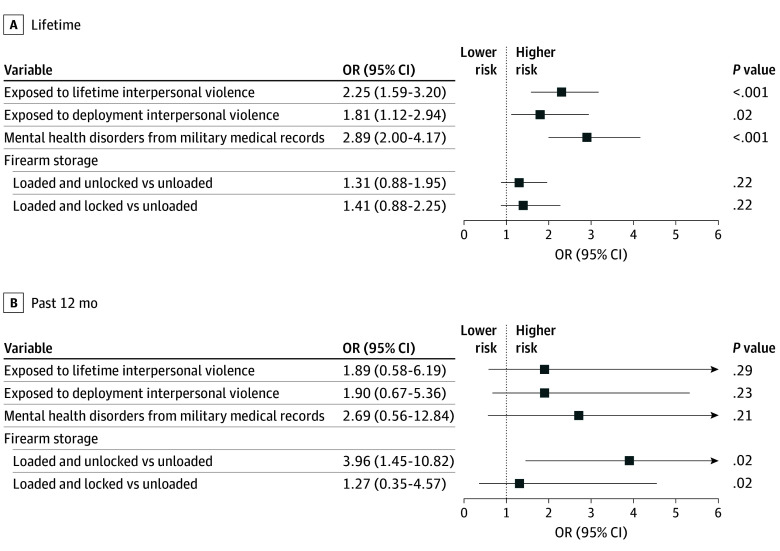
Forest Plots of Multivariable Logistic Model Results for Lifetime and 12-Month Suicide Attempt Among Firearm Owners (n = 6561) Multivariable models controlled for Army and demographic variables, including current military status, sex, marriage status, educational level, and military rank.

Unsecured storage was associated with 12-month suicide attempt (OR, 3.96; 95% CI, 1.45-10.82) compared with storing firearms securely (eg, unloaded). Carrying a firearm around the neighborhood was associated with 12-month suicide attempt (OR, 1.90; 95% CI, 0.80-4.49). Carrying types of weapons other than firearms around the neighborhood was associated with lifetime suicide ideation (OR, 1.32; 95% CI, 1.14-1.54) (eTable 6 in Supplement 1) and 12-month suicide attempt (OR, 10.42; 95% CI, 2.89-37.54) (Table 3 and Figure 2).

**Table 3.** Multivariable Associations of Sociodemographic, Army Career, and Mental Health Characteristics With 12-Month Suicide Attempt

Characteristic	12-mo Suicide attempt (N = 6561)^a^								
	Unsecured firearm storage			Firearm carrying			Carrying other weapons		
	OR (95% CI)	χ^2^	*P* value	OR (95% CI)	χ^2^	*P* value	OR (95% CI)	χ^2^	*P* value
Female sex	2.99 (0.89-10.01)	3.17	.07	2.83 (0.97-8.22)	3.67	.06	2.89 (0.96-8.65)	3.61	.06
Educational level (vs high school graduate)									
<High school or GED	2.51 (0.67-9.46)	3.46	.33	2.47 (0.60-10.12)	3.55	.31	2.98 (0.82-10.87)	4.14	.25
Some college	1.48 (0.37-6.00)			1.80 (0.44-7.33)			2.12 (0.51-8.85)		
≥College degree	0.51 (0.09-3.00)			0.53 (0.09-3.05)			0.62 (0.12-3.24)		
Current military status (vs active duty)									
Activated G or R or other	0.71 (0.06-7.73)	13.56	.004	0.68 (0.06-7.28)	12.52	.006	0.75 (0.07-8.31)	12.84	.005
G or R not currently on active duty	1.17 (0.16-8.63)			1.22 (0.17-8.71)			1.20 (0.15-9.40)		
Retired, separated Army, or G or R	6.52 (1.63-26.10)			6.57 (1.59-27.11)			6.66 (1.53-28.91)		
Marriage status (vs never married)									
Currently married	0.32 (0.07-1.41)	8.56	.01	0.32 (0.07-1.38)	8.11	.02	0.31 (0.07-1.25)	9.05	.01
Previously married	1.86 (0.49-7.00)			1.86 (0.47-7.41)			2.03 (0.56-7.31)		
Rank (vs E1-E4)^b^									
E5-E6	0.77 (0.26-2.33)	3.19	.36	0.82 (0.24-2.84)	3.59	.31	0.86 (0.27-2.75)	2.64	.45
E7-E9	0.47 (0.11-2.07)			0.45 (0.10-2.05)			0.51 (0.12-2.16)		
WO or CO	0.18 (0.02-1.38)			0.17 (0.02-1.26)			0.23 (0.03-1.73)		
Exposed to interpersonal violence									
Lifetime	1.89 (0.58-6.19)	1.12	.29	1.99 (0.62-6.41)	1.33	.25	1.98 (0.61-6.46)	1.29	.26
Deployment	1.90 (0.67-5.36)	1.46	.23	1.98 (0.67-5.80)	1.55	.21	1.85 (0.68-5.02)	1.47	.23
Mental health disorder diagnosis^c^	2.69 (0.56-12.84)	1.55	.21	2.52 (0.51-12.44)	1.31	.25	2.50 (0.54-11.55)	1.39	.24
Firearm storage (vs unloaded)									
Loaded and unlocked	3.96 (1.45-10.82)	7.44	.02	NA	NA	NA	NA	NA	NA
Loaded and locked	1.27 (0.35-4.57)			NA			NA		
Carrying weapons around the neighborhood all, most, or some vs none of the time									
Firearms	NA	NA	NA	1.90 (0.80-4.49)	2.15	.14	NA	NA	NA
Other (knife, mace, or club)	NA			NA	NA	NA	10.42 (2.89-37.54)	12.93	<.001

Abbreviations: CO, commissioned officer; G, Guard; GED, General Educational Development; NA, not applicable; OR, odds ratio; R, Reserve; WO, warrant officer.

^a^ Firearm storage, firearm carrying, and other weapon carrying were entered into each multivariable model separately. Analyses were weighted.

^b^ E1 to E4 indicates junior enlisted and E5 to E9, senior enlisted or noncommissioned officer.

^c^ Mental health disorder diagnoses were obtained from the military medical record.

## Discussion

In this retrospective cross-sectional study of military service members, unsecure firearm storage was associated with increased odds of suicidal ideation and attempt. Carrying a weapon other than a firearm around the neighborhood was also associated with increased odds of suicide ideation and attempt among firearm owners. These findings highlight the associations between firearm storage, firearm and weapon carrying, and suicide risk in a large sample of firearm-owning US Army soldiers.

Consistent with prior findings in military service member samples,^5,9^ unsecure firearm storage was associated with increased odds of 12-month suicide attempt and 12-month and lifetime suicide ideation among firearm-owning US Army soldiers, even after controlling for history of mental health disorders from the military medical record, exposure to interpersonal violence (both lifetime and during deployment), military career characteristics, and sociodemographic variables. This suggests that unsecure firearm storage may lead to heightened suicide risk, that heightened risk may lead to less secure firearm storage practices, or that there may be additional unmeasured variables accounting for these consistent associations across studies. In the case of the former 2 possibilities, secure storage interventions such as lethal means counseling^28,29^ may be useful tools to decrease risk of suicide death among firearm-owning US Army soldiers.

Contrary to prior research findings,^11^ firearm carrying around the neighborhood was not associated with history of suicidal behaviors. Given the lack of data on exactly when the suicide attempt occurred in the past year and when the individual began carrying a firearm, this finding is somewhat difficult to interpret. Due to the lethality of firearms as a suicide method,^30^ it is unlikely that any of these individuals had previously attempted suicide using a firearm. Carrying other types of weapons was associated with lifetime suicidal ideation, suggesting individuals at higher risk of suicidal behaviors may be in possession of other weapons in the home. One potential clinical interpretation of these data is that unsecured firearm storage and weapon carrying represent unique identifiers of suicide risk.

Specifically, the link between riskier firearm and weapon behaviors and suicide ideation and attempt may be related to an altered perception of threat. One study found that individuals who frequently carried handguns viewed the world as more dangerous, showed more shifts in anxiety and fear, and experienced prolonged anxiety and fear reactions.^31^ Such a prolonged negative affect may be linked to increased thoughts of suicide or a greater likelihood to act on suicidal thoughts.

Understanding of the relationship between firearm behaviors and suicide risk is evolving. Recent research highlights the consideration of several potentially important moderating or mediating variables in these relationships, including reasons for gun ownership, especially for safety and protection,^32^ and heightened threat perception.^31^ Research suggests that firearm owners who own firearms for self-protection exhibit higher rates of threat expectancy, suicidal ideation, and past-month suicide attempt than firearm owners who own firearms for reasons other than self-protection and individuals who do not own firearms.^33^ Additionally, individuals with high threat perception view secure firearm storage as ineffective for preventing suicide,^34^ as do those who store firearms unsecured.^35^ Thus, heightened threat perception may function as a catalyst for unsecure firearm storage, firearm and weapon carrying, and suicidal ideation and attempt among firearm owners. Further research is necessary to explore the possible moderating or mediating impact of this risk factor.

### Limitations

These results should be interpreted in the context of several study limitations. Because these are cross-sectional data, we were unable to determine causality between firearm storage and carrying behaviors and suicide ideation and attempt. Future STARRS-LS waves will allow for prospective evaluations. Findings in this study were based on surveys of living respondents, with few suicide deaths in the sample. Our data lacked information on suicide attempt methods. Given that firearm suicide attempts are highly lethal, as high as 90%,^30^ it is likely that few living respondents of firearm suicide attempts would be included among survey respondents. Symptoms of suicidal ideation and suicide attempts were based on self-report and were not confirmed by clinical assessment. Additionally, we did not assess threat perception in this study, but we did control for history of mental health disorders from the military medical record, including PTSD diagnosis, which involves hypervigilance for threat. Furthermore, these data reflect active-duty service members and recently discharged veterans, and findings may not be generalizable to civilians.

## Conclusions

In this retrospective cross-sectional study, unsecured firearm storage practices were significantly associated with increased suicide risk among US Army soldiers across all time frames, even after controlling for lifetime mental health disorders and history of SLE. Carrying a weapon other than a firearm was also associated with heightened suicide risk. These findings suggest additional directions for future study. Future STARRS-LSW2 data will study prospective associations between suicidal ideation and attempt and firearm behaviors. There is rich potential for further examination of the relationship between threat perception and anxiety and fear instability and how those characteristics relate to suicidal ideation and attempt. Future studies can also examine other potential environmental mechanisms by which geographic location, firearm laws, firearm behaviors, and suicidal behaviors are linked.

## Notes

Supplement 1. **eMethods.** Measures **eTable 1.** List of Stressful Life Events and Codes Categorized Thematically **eTable 2.** Frequencies and Weighted Percentages of the STARRS-LSW2 Sample **eTable 3.** Univariable Associations of Sociodemographic, Army Career, Firearm Storage, and Mental Health Characteristics With 30-Day, 12-Month, and LT Suicide Ideation **eTable 4.** Univariable Associations of Sociodemographic, Army Career, Firearm Storage, and Mental Health Characteristics With 12-Month and LT Suicide Attempt **eTable 5.** Multivariable Associations of Sociodemographic, Army Career, and Mental Health Characteristics With 12-Month Suicide Ideation **eTable 6. **Multivariable Associations of Sociodemographic, Army Career, and Mental Health Characteristics With LT Suicide Ideation **eTable 7.** Multivariable Associations of Sociodemographic, Army Career, and Mental Health Characteristics With LT Suicide Attempt

Supplement 2.
Data Sharing Statement
