# Supplementary material for: Firearm Storage and Carrying Practices and Suicidal Behaviors in US Army Service Members
Source: JAMA Netw Open. 2026 Apr 21;9(4):e268268. doi: 10.1001/jamanetworkopen.2026.8268 (PMC13100839; doi:10.1001/jamanetworkopen.2026.8268)
Supplement: Supplement 1. — eMethods. Measures eTable 1. List of Stressful Life Events and Codes Categorized Thematically eTable 2. Frequencies and Weighted Percentages of the STARRS-LSW2 Sample eTable 3. Univariable Associations of Sociodemographic, Army Career, Firearm Storage, and Mental Health Characteristics With 30-Day, 12-Month, and LT Suicide Ideation eTable 4. Univariable Associations of Sociodemographic, Army Career, Firearm Storage, and Mental Health Characteristics With 12-Month and LT Suicide Attempt eTable 5. Multivariable Associations of Sociodemographic, Army Career, and Mental Health Characteristics With 12-Month Suicide Ideation eTable 6. Multivariable Associations of Sociodemographic, Army Career, and Mental Health Characteristics With LT Suicide Ideation eTable 7. Multivariable Associations of Sociodemographic, Army Career, and Mental Health Characteristics With LT Suicide Attempt [file jamanetwopen-e268268-s001.pdf]

Supplementary Online Content

Dempsey CL, West JC, Houtsma C, et al. Firearm storage and carrying practices and suicidal behaviors in US Army service members. *JAMA Netw Open*. 2026;9(4):e268268. doi:10.1001/jamanetworkopen.2026.8268

eMethods. Measures

eTable 1. List of Stressful Life Events and Codes Categorized Thematically

eTable 2. Frequencies and Weighted Percentages of the STARRS-LSW2 Sample

eTable 3. Univariable Associations of Sociodemographic, Army Career, Firearm Storage, and Mental Health Characteristics With 30-Day, 12-Month, and LT Suicide Ideation

eTable 4. Univariable Associations of Sociodemographic, Army Career, Firearm Storage, and Mental Health Characteristics With 12-Month and LT Suicide Attempt

eTable 5. Multivariable Associations of Sociodemographic, Army Career, and Mental Health Characteristics With 12-Month Suicide Ideation

eTable 6. Multivariable Associations of Sociodemographic, Army Career, and Mental Health Characteristics With LT Suicide Ideation

eTable 7. Multivariable Associations of Sociodemographic, Army Career, and Mental Health Characteristics With LT Suicide Attempt

This supplementary material has been provided by the authors to give readers additional information about their work.

© 2026 Dempsey CL et al. *JAMA Network Open*.

eMethods. Measures

Suicidal Behaviors. The STARRS LS W2 survey assessed self-reported past month, past year and lifetime suicide ideation and history of past year and lifetime suicide attempt using the Columbia-Suicide Severity Rating Scale (C-SSRS) modified for Army STARRS.Recent and lifetime suicide ideation items included: *Since your last survey in (month/year), how often: a.) did you have thoughts of killing yourself; b.) wish you were dead or would go to sleep and never wake up; c.) think about how you might kill yourself, for example taking pills, shooting yourself, or work out a plan to kill yourself?; with response options including every day or nearly every day, 3 to 4 days per week, 1 to 2 days per week , 1 to 3 days a month, less than once a month or never. Age the first time you a:) had thoughts of killing yourself; b.) wished you were dead or would go to sleep and never wake up? About how old were you the very first time you thought about how you might kill yourself or work out a plan to kill yourself?*

Past year and lifetime suicide attempt items included*: Did you make a suicide attempt (i.e., purposefully hurt yourself with at least some intention to die at any time since the last survey? About how old were you when you made the first attempt since the last survey? When was your most recent attempt?* Dichotomous variables (yes/no) were created for past month suicide ideation, past year suicide ideation, lifetime suicide ideation, past year suicide attempt and lifetime suicide attempt.

© 2026 Dempsey CL et al. *JAMA Network Open*.

eTable 1: List of stressful life events and codes categorized thematically

**Lifetime Stressful Events**	
**Type**	**Survey Questions**
**Accident**	Life-threatening accident where you escaped injury
	Any other experiences that put you at risk of death or serious injury
	Life-threatening illness
	Life-threatening injury
	Serious injury or unexpected death of a close loved one?
	Witnessed someone being seriously injured/killed
Exposure	Exposed to details of highly stressful events as part of job
	Discovered or handled a dead body
Interpersonal Violence	Serious Physical assault
	Sexual Assault or Rape
Other	Something else
**Lifetime Deployment Stressors**	
**Type**	**Survey Questions**
Combat Stress	Have direct responsibility for the death of US or ally
	Have a close call
	Go on combat patrols or have other dangerous duty
	Have direct responsibility for death of an enemy combatant
	Exposed to sight/sounds/smells/ of severely wounded or dying/see dead bodies
	Fire rounds at the enemy or take enemy fire
	Get wounded by the enemy
	Have direct responsibility for the death of a non-combatant
	Save the life of a servicemember or civilian
	Other
Interpersonal violence	Get bullied/hazed by 1+ members of unit
	Get seriously physically assaulted
	Get sexually assaulted or raped
Atrocities	See homes or villages that have been destroyed or people begging for food
	Witness violence within local people or mistreatment of non-combatant

© 2026 Dempsey CL et al. *JAMA Network Open*.

eTable 2: Frequencies and Weighted Percentages of the **STARRS-LSW2 Sample** [a]

**Characteristic**	**Total Sample (*n* =12,022)**	
	n	(W%) [b]
**Sex**		
Female	1926	16.4
Male	10096	83.6
**Military Status**		
Active Duty	3456	27.5
Activated G/R or other	535	3.4
G/R not currently on Active Duty	2348	16.9
Retired, Separated Army, G/R	5683	52.2
**Race**		
White	8316	65.2
Black	1516	16.1
Hispanic	1405	11.9
Asian/Other	785	6.8
**Rank**		
E1-E4	4621	42.1
E5-E6	4414	34.3
E7-E9	1220	9.7
WO/CO	1767	13.9
**Mental Health Disorder Diagnosis** [c]		
No	6725	55.6
Yes	5297	44.4
**Lifetime Interpersonal Violence**		
Never	9078	77.9
Ever	2944	22.1
**Deployment Interpersonal Violence**		
Never	10931	93.2
Ever	1091	6.8
**Firearm Owner**		
No	5461	45.4
Yes	6561	54.5
**Firearms storage** [d]		
Unloaded	1851	14.5
Loaded and unlocked	1447	11.8
Loaded and locked	3238	46.5
**Firearm carrying in the neighborhood**		
None of the time	3183	26.1
All/Most/Some	3381	27.5
**Weapon carrying (knife, mace or club) in the neighborhood**		
None of the time	3020	25.3
All/Most/Some	3542	28.3

Notes:
Due to rounding, total percentages may not round to 100%
Data are based on records from concurrent month and year of Army records or last administrative record and supplemented by survey data
[a] LSW2 total sample who agreed to administrative linkage.
[b] Percentages are weighted (W%)
[c] Mental health disorders are from the military medical record
[d] Firearm owners (N = 6,561) were asked the firearm storage and carrying practices and other weapon questions

© 2026 Dempsey CL et al. *JAMA Network Open*.

eTable 3: Univariable associations of sociodemographic, Army career, firearm storage, and mental health characteristics with 30-day, 12-month, and LT suicide ideation (N = 6,561)

Characteristic	30-day Suicide Ideation		12-month Suicide Ideation		LT Suicide Ideation	
	OR	(95% CI)	OR	(95% CI)	OR	(95% CI)
**Demographics**						
**Sex**						
Female vs. Male	1.80	(1.34, 2.42)	1.56	(1.23, 2.00)	1.43	(1.18, 1.72)
χ^2, p_{fdr} [a]	15.19, <.001		13.00, <.001		13.75, <.001	
**Deployment**						
Current/Previous vs. Never	1.48	(1.13, 1.95)	1.39	(1.09, 1.76)	1.16	(0.96, 1.41)
χ^2, p_{fdr}	8.01, .007		7.23, .010		2.50, .142	
**Current Military Status**						
Activated G/R or other vs. Active Duty	1.12	(0.57, 2.19)	0.98	(0.60, 1.61)	1.18	(0.84, 1.65)
G/R not currently on Active Duty vs. Active Duty	1.60	(1.03, 2.49)	1.61	(1.16, 2.24)	1.44	(1.14, 1.80)
Retired, Separated Army, G/R vs. Active Duty	3.42	(2.30, 5.08)	2.96	(2.15, 4.08)	2.49	(2.09, 2.97)
χ^2, p_{fdr}	55.27, <.001		57.99, <.001		120.51, <.001	
**Marriage Status**						
Currently married vs. Never married	0.77	(0.57, 1.03)	0.70	(0.55, 0.91)	0.68	(0.57, 0.81)
Previously married vs. Never married	1.41	(0.99, 2.02)	1.29	(0.99, 1.69)	1.41	(1.06, 1.86)
χ^2, p_{fdr}	21.12, <.001		29.34, <.001		61.46, <.001	
**Rank**						
E5-E6 vs. E1-E4	0.63	(0.49, 0.82)	0.65	(0.52, 0.82)	0.60	(0.48, 0.74)
E7-E9 vs. E1-E4	0.48	(0.32, 0.72)	0.44	(0.32, 0.61)	0.46	(0.34, 0.62)
WO/CO vs. E1-E4	0.42	(0.28, 0.63)	0.40	(0.30, 0.54)	0.44	(0.34, 0.56)
χ^2, p_{fdr}	26.84, <.001		47.98, <.001		67.42, <.001	
**Mental Health Disorder Diagnosis** [b]						
Yes vs. No	2.66	(2.13, 3.31)	2.38	(1.95, 2.91)	2.49	(2.04, 3.03)
χ^2, p_{fdr} [b]	75.58, <.001		73.23, <.001		81.77, <.001	
**Weapons**						
**Firearms storage**						
Loaded and unlocked vs. Unloaded	1.63	(1.27, 2.10)	1.61	(1.27, 2.03)	1.44	(1.22, 1.70)
Loaded and locked vs. Unloaded	1.27	(0.94, 1.70)	1.11	(0.86, 1.42)	0.94	(0.78, 1.14)
χ^2, p_{fdr}	14.61, .002		19.47, <.001		27.79, <.001	
**Firearm carrying in the neighborhood**						
All/Most/Some vs. None of the time	0.91	(0.74, 1.12)	0.98	(0.83, 1.16)	0.97	(0.84,1.13)
χ^2, p_{fdr}	0.74, .390		0.04, .839		0.14, .712	
**Weapon carrying (knife, mace, club) in the neighborhood**						
All/Most/Some vs. None of the time	1.35	(1.10, 1.67)	1.41	(1.16, 1.72)	1.52	(1.32, 1.74)
χ^2, p_{fdr}	8.14, .004		11.91, <.001		34.94, <.001	

Notes:
[a] FDR p-values presented
[b] Mental health disorder diagnosis from the military medical record.
Abbreviations: CO; Commissioned Officer, CI; 95% Confidence Intervals, FDR; False Discovery Rate, G; Guard, GED; General Educational Development, OR; odds ratio, R; Reserve, WO; Warrant Officer.
Reference after the vs.

© 2026 Dempsey CL et al. *JAMA Network Open*.

eTable 4: Univariable associations of sociodemographic, Army career, firearm storage, and mental health characteristics with 12-month and LT Suicide Attempt (*N* = 6561)

Characteristic	12-Month Suicide Attempt		LT Suicide Attempt	
	**OR**	**(95% CI)**	**OR**	**(95% CI)**
**Demographics**				
**Sex**				
Female vs. Male	3.47	(1.26, 9.58)	1.70	(1.18, 2.45)
χ^2, p_{fdr} [b]	5.81, .015		8.17, .006	
**Education**				
<High School + AltEd + GED vs. High school	3.78	(1.15, 12.44)	1.44	(0.91, 2.29)
Some college vs. High school	0.43	(0.09, 1.95)	0.51	(0.23, 1.15)
College+ vs. High school	0.16	(0.03, 0.78)	0.30	(0.17, 0.51)
χ^2, p_{fdr}	13.28, .008		33.44, <.001	
**Current Military Status**				
Activated G/R or other vs. Active Duty	1.02	(0.10, 10.44)	1.16	(0.41, 3.30)
G/R not currently on Active Duty vs. Active Duty	2.27	(0.34, 15.10)	1.46	(0.90, 2.38)
Retired, Separated Army, G/R vs. Active Duty	16.86	(4.47, 63.57)	3.18	(1.99, 5.10)
χ^2, p_{fdr}	26.07, <.001		28.69, <.001	
**Marriage Status**				
Currently married vs. Never married	0.30	(0.09, 1.02)	**0.57**	**(0.39, 0.84)**
Previously married vs. Never married	2.88	(0.89, 9.32)	1.34	(0.79, 2.27)
χ^2, p_{fdr}	14.60, .002		20.55, <.001	
**Rank**				
E5-E6 vs. E1-E4	0.41	(0.13, 1.26)	0.47	(0.33, 0.66)
E7-E9 vs. E1-E4	0.20	(0.05, 0.74)	0.33	(0.17, 0.62)
WO/CO vs. E1-E4	0.02	(0.01, 0.09)	0.21	(0.09, 0.46)
χ^2, p_{fdr}	31.28, <.001		34.01, <.001	
**Active service time**				
1-4 Years vs. 5-10 Years	2.24	(0.78, 6.45)	1.67	(1.14, 2.44)
>10 Years vs. 5-10 Years	0.21	(0.06, 0.73)	0.77	(0.51, 1.18)
χ^2, p_{fdr}	18.41, <.001		13.70, .002	
**Mental Health Disorder Diagnoses[a]**				
Yes vs. No	4.19	(1.14, 15.36)	3.40	(2.46, 4.69)
χ^2, p_{fdr}	4.69, .043		55.42, <.001	
**Firearms storage**				
Loaded and unlocked vs. Unloaded	4.93	(1.8, 13.51)	1.55	(1.04, 2.32)
Loaded and locked vs. Unloaded	1.66	(0.47, 5.89)	1.56	(0.97, 2.51)
χ^2, p_{fdr}	9.74, .011		6.45, .039	
**Firearm carrying in the neighborhood**				
All/Most/Some vs. None of the time	2.02	(0.72, 5.65)	1.12	(0.81, 1.56)
χ^2, p_{fdr}	1.82, .117		0.50, .479	
**Weapon carrying (knife, mace or club) in the neighborhood**				
All/Most/Some vs. None of the time	12.04	(3.61, 40.09)	1.43	(0.95, 2.15)
χ^2, p_{fdr}	16.55, <.001		2.93, .087	

Notes:

[a]FDR p-values presented

[b] Mental health disorder diagnosis from the military medical record.

Abbreviations: CO; Commissioned Officer, CI; 95% Confidence Intervals, FDR; False Discovery Rate, G; Guard, GED; General Educational Development, OR; odds ratio, R; Reserve, WO; Warrant Officer.

Reference after the vs.

© 2026 Dempsey CL et al. *JAMA Network Open*.

eTable 5: Multivariable associations of sociodemographic, Army career, and mental health characteristics with 12-month suicide ideation (N = 6,561)

Characteristic	12-Month Suicide Ideation					
	Firearm Storage [a]		Firearm Carry		Weapons Carry	
	OR	(95% CI)	OR	(95% CI)	OR	(95% CI)
**Sex**						
Female vs. Male	1.27	(0.97, 1.68)	1.20	(0.91, 1.58)	1.25	(0.95, 1.66)
χ^2, *p*	2.99, .08		1.61, .204		2.51, .11	
**Education**						
<High School/Alt/GED vs. High School	1.34	(1.00, 1.79)	1.36	(1.02, 1.81)	1.33	(0.99, 1.77)
Some college vs. High School	1.14	(0.83, 1.58)	1.17	(0.85, 1.61)	1.17	(0.85, 1.61)
College+ vs. High School	0.82	(0.56, 1.20)	0.81	(0.54, 1.20)	0.83	(0.56, 1.23)
χ^2, *p*	6.34, .10		7.16, .066		6.37, .10	
**Military Status**						
Activated G/R or other vs. Active Duty	0.94	(0.57, 1.56)	0.97	(0.58, 1.61)	0.94	(0.57, 1.56)
G/R not currently on Active Duty vs. Active Duty	1.37	(0.97, 1.93)	1.41	(1.00, 1.99)	1.41	(1.00, 1.98)
Retired, Separated Army, G/R	2.11	(1.49, 2.99)	2.14	(1.51, 3.05)	2.14	(1.51, 3.05)
χ^2, *p*	25.14, <.001		24.05, <.001		25.01, <.001	
**Marriage Status**						
Currently vs. Never married	0.77	(0.57, 1.03)	0.77	(0.57, 1.03)	0.76	(0.56, 1.02)
Previously vs. Never married	1.10	(0.78, 1.53)	1.12	(0.80, 1.55)	1.10	(0.79, 1.53)
χ^2, *p*	10.23, .01		10.56, .005		10.71, .005	
**Rank**						
E5-E6 vs. E1-E4	0.91	(0.72, 1.13)	0.90	(0.71, 1.13)	0.91	(0.73, 1.14)
E7-E9 vs. E1-E4	0.58	(0.43, 0.78)	0.58	(0.43, 0.78)	0.60	(0.44, 0.81)
WO/CO vs. E1-E4	0.79	(0.51, 1.23)	0.77	(0.49, 1.19)	0.80	(0.52, 1.24)
χ^2, *p*	12.80, .01		13.12, .004		10.77, .01	
**Lifetime Interpersonal Violence**						
Yes vs. No	1.65	(1.31, 2.08)	1.70	(1.35, 2.14)	1.65	(1.31, 2.08)
χ^2, *p*	17.86, <.001		20.20, <.001		17.88, <.001	
**Deployment Interpersonal Violence**						
Yes vs. No	1.67	(1.19, 2.34)	1.69	(1.22, 2.34)	1.68	(1.22, 2.33)
χ^2, *p*	9.06, .01		10.03, .002		9.98, .002	
**Mental Disorder Diagnosis [b]**						
Yes vs. No	2.11	(1.69, 2.64)	2.07	(1.65, 2.59)	2.05	(1.64, 2.56)
χ^2, *p*	43.32, <.001		40.16, <.001		40.46, <.001	
**Firearm Storage**						
Loaded/Unlocked vs. Unloaded	1.44	(1.12, 1.84)				
Loaded/Locked vs. Unloaded	1.01	(0.78, 1.29)				
χ^2, *p*	12.06, .002					
**Carrying Firearms Around the Neighborhood**						
All/Most/Some vs. None of the time			0.89	(0.74, 1.06)		
χ^2, *p*			1.72, .190			
**Weapon Carrying (knife, mace, or club) Around the Neighborhood**						
All/Most/Some vs. None of the time					1.22	(0.98, 1.51)
χ^2, *p*					3.30, .07	

Notes:
[a] Firearm storage, firearm carrying and weapon carrying entered into each MVA model separately
[b] Mental health diagnoses are from the military medical record
Abbreviations: CO; Commissioned Officer, CI; 95% Confidence Intervals, G; Guard, GED; General Educational Development, OR; odds ratio, R; Reserve, WO; Warrant Officer. Reference after the vs.

© 2026 Dempsey CL et al. *JAMA Network Open*.

eTable 6: Multivariable associations of sociodemographic, Army career, and mental health characteristics with LT suicide ideation (N= 6,561)

Characteristic	Lifetime Suicide Ideation					
	Firearm Storage [a]		Firearm Carry		Weapons Carry	
	**OR**	**(95% CI)**	**OR**	**(95% CI)**	**OR**	**(95% CI)**
**Sex**						
Female vs. Male	1.07	(0.85, 1.35)	1.01	(0.80, 1.28)	1.07	(0.85, 1.35)
χ^2, *p*	0.35, .552		0.01, .941		0.36, .549	
**Education**						
<High School/Alt/GED vs. High School	1.34	(1.05, 1.70)	1.35	(1.06, 1.73)	1.33	(1.04, 1.70)
Some college vs. High School	0.94	(0.75, 1.19)	0.96	(0.76, 1.22)	0.96	(0.76, 1.22)
College+ vs. High School	1.15	(0.88, 1.52)	1.14	(0.87, 1.50)	1.17	(0.90, 1.53)
χ^2, *p*	6.85, .076		6.51, .089		6.14, .105	
**Current Military Status**						
Activated G/R or other vs. Active Duty	1.12	(0.78, 1.63)	1.16	(0.80, 1.67)	1.13	(0.78, 1.64)
G/R not currently on Active Duty vs. Active Duty	1.20	(0.92, 1.57)	1.22	(0.94, 1.60)	1.23	(0.94, 1.61)
Retired, Separated Army, G/R	1.72	(1.41, 2.11)	1.74	(1.42, 2.12)	1.75	(1.43, 2.15)
χ^2, *p*	33.94, <.001		33.08, <.001		34.14, <.001	
**Marriage Status**						
Currently vs. Never Married	0.73	(0.60, 0.89)	0.74	(0.60, 0.90)	0.73	(0.59, 0.89)
Previously vs. Never Married	1.24	(0.88, 1.73)	1.29	(0.92, 1.79)	1.26	(0.90, 1.76)
χ^2, p	27.73, <.001		29.50, <.001		30.08, <.001	
**Rank**						
E5-E6 vs. E1-E4	0.74	(0.61, 0.91)	0.74	(0.60, 0.91)	0.75	(0.61, 0.93)
E7-E9 vs. E1-E4	0.50	(0.37, 0.68)	0.51	(0.38, 0.69)	0.53	(0.39, 0.72)
WO/CO vs. E1-E4	0.55	(0.39, 0.78)	0.54	(0.38, 0.77)	0.58	(0.40, 0.82)
χ^2, *p*	25.36, <.001		25.49, <.001		21.81, <.001	
**Lifetime Interpersonal Violence**						
Yes vs. No	1.99	(1.67, 2.38)	2.06	(1.73, 2.45)	1.97	(1.65, 2.36)
χ^2, *p*	58.78, <.001		66.97, <.001		56.02, <.001	
**Deployment Interpersonal Violence**						
Yes vs. No	1.91	(1.45, 2.52)	1.91	(1.47, 2.49)	1.91	(1.46, 2.50)
χ^2, *p*	21.50, <.001		23.33, <.001		22.31, <.001	
**Mental Health Disorder Diagnosis** [b]						
Yes vs. No	2.34	(1.88, 2.90)	2.30	(1.85, 2.87)	2.29	(1.84, 2.83)
χ^2, *p*	59.21, <.001		55.52, <.001		57.26, <.001	
**Firearm Storage**						
Loaded/Unlocked vs. Unloaded	1.28	(1.07, 1.53)				
Loaded/Locked vs. Unloaded	0.86	(0.71, 1.03)				
χ^2, *p*	17.85, <.001					
**Carrying Firearms Around the Neighborhood**						
All/Most/Some vs. None of the time			0.86	(0.73, 1.02)		
χ^2, *p*			3.08, .079			
**Weapon Carrying (knife, mace, or club) Around the Neighborhood**						
All/Most/Some vs. None of the time					1.32	(1.14, .54)
χ^2, *p*					13.13, <.001	

Notes:
[a] Firearm storage, firearm carrying and weapon carrying entered into each MVA model separately
[b] Mental health disorder diagnosis from the military medical record
Abbreviations: CO; Commissioned Officer, CI; 95% Confidence Intervals, G; Guard, GED; General Educational Development, OR; odds ratio, R; Reserve, WO; Warrant Officer. Reference after the vs.

© 2026 Dempsey CL et al. *JAMA Network Open*.

eTable 7: Multivariable associations of sociodemographic, Army career, and mental health characteristics with LT suicide attempt (N = 6,561)

Characteristic	Lifetime Suicide Attempt					
	Firearm Storage [a]		Firearm Carrying		Weapons Carrying	
	OR	(95% CI)	OR	(95% CI)	OR	(95% CI)
**Sex**						
Female vs. Male	1.28	(0.85, 1.91)	1.19	(0.79, 1.81)	1.21	(0.80,1.82)
χ^2, p	1.39, .238		0.70, .403		0.84, 0.360	
**Education**						
<High School/Alt/GED vs. High School	1.23	(0.75, 2.00)	1.23	(0.76, 1.99)	1.24	(0.77, 2.01)
Some college vs. High School	0.69	(0.43, 1.12)	0.70	(0.43, 1.13)	0.70	(0.43, 1.14)
College+ vs. High School	0.62	(0.37, 1.02)	0.60	(0.36, 1.00)	0.61	(0.36, 1.02)
χ^2, p	7.21, .065		7.15, .067		7.19, 0.066	
**Military Status**						
Activated G/R or other vs. Active Duty	1.02	(0.35, 2.96)	1.06	(0.37, 3.06)	1.05	(0.36, 3.05)
G/R not currently on Active Duty vs. Active Duty	0.95	(0.56, 1.61)	0.96	(0.57, 1.63)	0.96	(0.57, 1.63)
Retired, Separated Army, G/R vs. Active Duty	1.62	(0.99, 2.66)	1.65	(1.01, 2.69)	1.65	(1.01, 2.69)
χ^2, p	7.48, .058		7.53, .056		7.52, 0.056	
**Marital Status**						
Currently vs. Never married	0.63	(0.42, 0.97)	0.65	(0.42, 1.01)	0.65	(0.42, 1.01)
Previously vs. Never married	0.98	(0.52, 1.83)	1.07	(0.55, 2.06)	1.06	(0.56, 2.02)
χ^2, p	8.13, .017		8.83, .012		9.09, .010	
**Rank**						
E5-E6 vs. E1-E4	0.64	(0.44, 0.94)	0.63	(0.43, 0.92)	0.63	(0.42, 0.93)
E7-E9 vs. E1-E4	0.45	(0.27, 0.75)	0.44	(0.26, 0.73)	0.44	(0.26, 0.74)
WO/CO vs. E1-E4	0.49	(0.20, 1.16)	0.47	(0.20, 1.14)	0.48	(0.19, 1.17)
χ^2, p	12.18, .006		12.84, .005		11.74, .008	
**Lifetime Interpersonal Violence**						
Yes vs. No	2.25	(1.59, 3.20)	2.38	(1.69, 3.35)	2.37	(1.66, 3.37)
χ^2, p	20.77, <.001		24.78, <.001		23.04, <.001	
**Deployment Interpersonal Violence**						
Yes vs. No	1.81	(1.12, 2.94)	1.79	(1.11, 2.91)	1.78	(1.11, 2.88)
χ^2, p	5.87, .02		5.68, .017		5.70, .016	
**Mental Health Disorder Diagnosis [b]**						
Yes vs. No	2.89	(2.00, 4.17)	2.88	(1.99, 4.16)	2.87	(1.98, 4.16)
χ^2, p	32.51, <.001		31.76, <.001		31.24, <.001	
**Firearm Storage**						
Loaded/Unlocked vs. Unloaded	1.31	(0.88, 1.95)				
Loaded/Locked vs. Unloaded	1.41	(0.88, 2.25)				
χ^2, p	3.05, .217					
**Carrying Firearms Around the Neighborhood**						
All/Most/Some vs. None of the time			0.96	(0.66, 1.39)		
χ^2, p			0.06, .814			
**Weapon Carrying (knife, mace, or club) Around the Neighborhood**						
All/Most/Some vs. None of the time					1.07	(0.70, 1.65)
χ^2, p					0.11, 0.742	

Notes:

[a] Firearm storage, firearm carrying and weapon carrying entered into each MVA model separately

[b] Mental health disorder diagnosis from the military medical record

Abbreviations: CO; Commissioned Officer, CI; 95% Confidence Intervals, G; Guard, GED; General Educational Development, OR; odds ratio, R; Reserve, WO; Warrant Officer. Reference after the vs..

© 2026 Dempsey CL et al. *JAMA Network Open*.
