# Supplementary material for: Firearm Storage and Carrying Practices and Suicidal Behaviors in US Army Service Members
Source: JAMA Netw Open. 2026 Apr 21;9(4):e268268. doi: 10.1001/jamanetworkopen.2026.8268 (PMC13100839; doi:10.1001/jamanetworkopen.2026.8268)
Supplement: Supplement 2. — Data Sharing Statement [file jamanetwopen-e268268-s002.pdf]

Data Sharing Statement

Dempsey. Firearm Storage and Carrying Practices and Suicidal Behaviors in US Army Service Members. *JAMA Netw Open*. Published April 21, 2026.
doi:10.1001/jamanetworkopen.2026.8268

Data
Data available: Yes
Data types: Deidentified participant data, Data dictionary
How to access data: https://www.icpsr.umich.edu/web/ICPSR/studies/35197
When available: With publication

Supporting Documents
Document types: None

Additional Information
Who can access the data: The Army Study to Assess Risk and Resilience in Servicemembers (STARRS) is restricted from general dissemination. Users interested in obtaining these data must complete a Restricted Data Use Agreement, specify the reasons for the request, and obtain IRB approval or notice of exemption for their research.
Types of analyses: Applicants may request access via Secure Dissemination or via ICPSR's Virtual Data Enclave (VDE). Those requesting VDE access will be required to pay an access fee.
Mechanisms of data availability: A signed data access agreement is required.
